# #MissingInAction: A review of the social media footprint of South African interventional radiology practitioners

**DOI:** 10.4102/jcmsa.v3i1.97

**Published:** 2025-03-12

**Authors:** Siviwe S. Mpateni, Kuhle M. Zwakala, Richard D. Pitcher, Michelle Da Silva

**Affiliations:** 1 Department of Radiodiagnosis, Faculty of Health Sciences, Stellenbosch University, Cape Town, South Africa; 2 Department of Marketing Management, Faculty of Business and Management Sciences, Cape Peninsula University of Technology, Cape Town, South Africa

**Keywords:** interventional radiology, social media, public health, medical education, health promotion, South Africa

## Abstract

**Background:**

Interventional radiology (IR) is a rapidly developing branch of medicine; however, the general awareness of the subspeciality among patients and medical colleagues is limited. Social media (SM) has become an integral part of information transfer globally, and its utility as an effective communication tool can be leveraged by IRs to bridge this knowledge gap. This study investigated the SM footprint of IR providers in South Africa.

**Methods:**

An online analysis of radiology practices (*N* = 100) registered on the Radiological Society of South Africa (RSSA) website was performed. The SM footprint of practices offering IR services was audited, and statistical analysis was performed to examine the level of SM uptake, the relationship between SM uptake in urban versus non-urban locations and between practice size.

**Results:**

There were 38 practices offering IR services with 68% (*n* = 26) located in major metropolitan areas. A systematic online Google search revealed that 84% (*n* = 32) had a website and the most widely used SM platform was Facebook. There was a statistically significant correlation between the size of the radiology practices and the total number of SM accounts (*p* < 0.05).

**Conclusion:**

Most South African IR practitioners have a SM presence with larger radiology practices establishing a broad digital presence on SM. Poor SM visibility, particularly from smaller practices, on these platforms may limit their ability to reach their target audience.

**Contribution:**

Improving the current usage of SM by IR practitioners may present an opportunity to display their services.

## Introduction

The turn of the millennium saw a rapid transition in the way the internet was used from Web 1.0 to Web 2.0 allowing individual internet users to evolve from passive viewers to contributors.^1^ This transition from ‘read-only’ Internet 1.0 to the more ‘wildly read-write’ Internet 2.0 was a milestone in the history of the Internet, and this environment facilitated the development of digital social networks and platforms to exchange information.^2^

Social media (SM) are digital interfaces through which individuals and institutions can create and share information in real time.^3^ Social media has been described as one of the three major technological revolutions that have taken place in this century and has transformed digital communication.^2^ The rise of SM has rapidly placed it as an integral part of social and professional discourse across the world, which has ushered in an unprecedented level of audience interaction and elevated the level of engagement between audience members and each other.^4^ Furthermore, the low cost of participation and wide reach have made SM a powerful tool for the rapid dissemination of information. These characteristics present infinite potential in medicine for patient education, interaction with clinical colleagues and engaging with trainees.^5,6^ The utility of SM has proven to be an effective tool for rapid communication in healthcare.^7^

It is recognised that the ubiquity of the internet and SM has changed how medicine is practiced around the world.^1^ This has transformed the utility of SM from simple information-sharing functions to a means of addressing public health challenges, healthcare quality, disaster preparedness and pandemic surveillance.^7^ Considering these factors, SM presents an abundance of opportunity in the South African healthcare sector for patient engagement, education and enhancing the visibility of emerging medical disciplines.

Interventional radiology (IR) is a new and rapidly evolving sub-specialty of radiology that encompasses a wide range of minimally invasive image-guided diagnostic and therapeutic procedures in nearly every human organ system.^8,9,10^ These minimally invasive image-guided techniques allow interventional radiologists to navigate wires and catheters to almost any place in the body to arrest active haemorrhage, open blocked arteries in peripheral artery disease, extract clots in acute stroke patients and perform precise delivery of chemotherapeutic agents in cancer patients, among others. These procedures are often lifesaving, significantly less invasive than their surgical alternative and can present alternative care options for patients unfit for surgery.^11^

Unfortunately, general awareness and education about IR among patients, medical trainees and medical colleagues has been proven to be limited.^8^ This lack of public awareness of the specialty is observed by Baerlocher and colleagues with as low as 6% of patients in their cohort being aware of the field of IR.^10^ However, despite the specialty’s relative obscurity, the impact of IR on healthcare systems worldwide has reduced healthcare costs, improved patient outcomes and satisfaction.^12^

Social media holds immense potential for the South African IR fraternity to promote the sub-specialty and improve awareness of the expanding capabilities and breadth of procedures available to their respective communities.^13^ In a survey evaluating the public awareness of IR, most respondents preferred to learn more about the specialty by viewing short educational videos on websites such as YouTube.^11^

There are currently no IR Fellowship training opportunities in South Africa and expanding SM presence can allow IRs to share novel procedures, raise public awareness and advertise their clinical practice.^13^ Unfortunately, there is currently no research into the use of SM in the South African IR community.

This study aims to examine the SM footprint of IR practitioners in South Africa and analyse any potential relationship between practice-related factors (i.e. geographic location and practice size) with SM uptake by IR practices in South Africa.

## Research methods and design

We performed an online audit of the practices listed on the Radiological Society of South Africa (RSSA) website, tabulated the practice location within the nine South African provinces and 8 metropolitan municipalities, the number of radiologists, private or public sector, the number of branches, availability of an IR or angiography service, website and presence on SM platforms, namely Facebook, X (formerly Twitter), LinkedIn, Instagram and YouTube.

We use descriptive and comparative statistical methods to achieve the following:

- An audit of the radiology practices and academic institutions registered under the RSSA website and offering IR services and their presence on five SM platforms, namely Facebook, YouTube, Instagram, X (formerly Twitter), and LinkedIn.
- Analysis of the association between practice-related factors (geographical location and practice size) and their SM footprint.

### Study population

The RSSA is the Professional Association of Radiologists in South Africa with a membership base of close to 900 individual radiologists and 100 practices, which are listed on their website (https://rssa.co.za/radiology-practices-in-sa/). This public platform provides detailed information on each listed practice providing the practice address, names of practicing radiologists and the services offered. We audited 100 public and private sector radiology practices, and all practices that offered IR or angiography as a service in at least one of their branches (*N* = 38) were analysed, and a systematic Google search for their websites was performed.

### Data collection

Data were tabulated using Microsoft Excel 2016 (Microsoft, Redmond, WA, United States [US]).

### Website analysis

A Google search was performed for each radiology practice offering IR or angiography in at least one of their branches in South Africa. This identified any practice or institutional website, and each website was assessed for any links to the five SM accounts analysed.

### Social media analysis

Social media links to Facebook, X (formerly Twitter), LinkedIn, Instagram and YouTube identified on each practice website were tabulated.

### Data analysis

Statistical analysis was performed using SPSS (IBM, New York, NY, US). *P < 0.05 was considered statistical significance.* Population statistics of all South African provinces were obtained from the Department of Statistics of South Africa Mid-Year population estimates 2022.^14^ The urban location of practices was defined as practice branches within the eight metropolitan municipalities (Buffalo City, City of Cape Town, Ekurhuleni, City of eThekwini, City of Johannesburg, Mangaung, Nelson Mandela Bay and the City of Tshwane) as defined by Department of Cooperative Governance of South Africa. The Mann–Whitney test was used to analyse the relationship between SM uptake in urban vs non-urban practices and the Spearman’s correlation test was used to analyse the relationship between practice size and SM uptake.

### Ethical considerations

Ethical clearance to conduct this study was obtained from the Stellenbosch University Faculty of Health Sciences Health Research Ethics Committee (No. S21/11/232).

## Results

There were 100 radiology practices registered on the RSSA website in November 2023 with 89 of these being private practices and 11 public institutions. Most radiology practices were in the major metropolitan areas (67.4%) with the Gauteng province having the highest number (*n* = 45) of general radiology practices. Mpumalanga and the Northern Cape provinces had the least number with one practice each (Table 1).

**TABLE 1:** Distribution of South African IR practices by province.

Province	Population (million)	Radiologists	General radiology practices	Number of IR practices	Population/IR (million)
Gauteng	16.1	237	45	16	1.00
KZN	11.5	86	16	8	1.44
Western Cape	7.2	127	14	7	1.02
Eastern Cape	6.7	31	6	2	3.35
Limpopo province	5.9	6	5	2	2.95
Mpumalanga	4.7	8	1	0	-
North West	4.2	13	4	2	2.10
Free State	2.9	23	8	1	2.90
Northern Cape	1.3	5	1	0	-

IR, Interventional radiology; KZN, KwaZulu-Natal.

**FIGURE 1:**
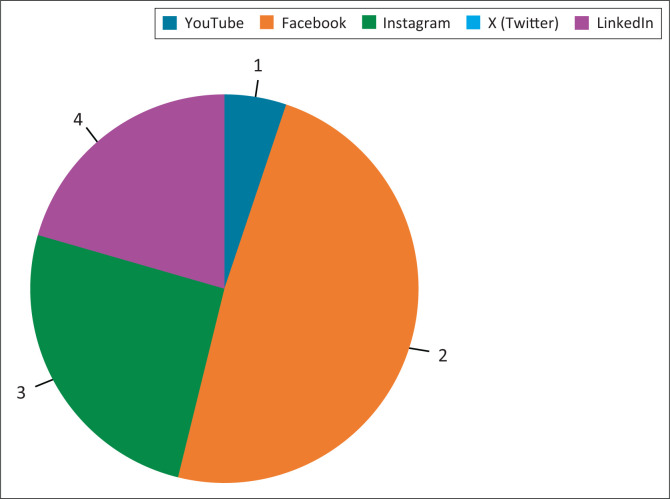
Distribution of interventional radiology social media accounts.

There were 38 practices that had listed IR or angiography as services offered in at least one of their branches. Across South Africa, there was one IR practice for every 1 594 737 people with Gauteng having the lowest IR-population ratio and the Eastern Cape province had the highest ratio of 1 IR for 3.35 million people (Table 1). There were no IR practitioners identified in the Mpumalanga and Northern Cape provinces. Most IR practices (*n* = 32, 84.2%) had a business website and only 15.8% of these practices did not have a presence on any of the analysed SM platforms. The most prevalent form of SM was Facebook (50%), followed by Instagram (26.3%) and LinkedIn (21.1%). None of the analysed practice websites had a link to a X (formerly Twitter) account and only 5.3% had a YouTube account (Figure 1).

**FIGURE 2:**
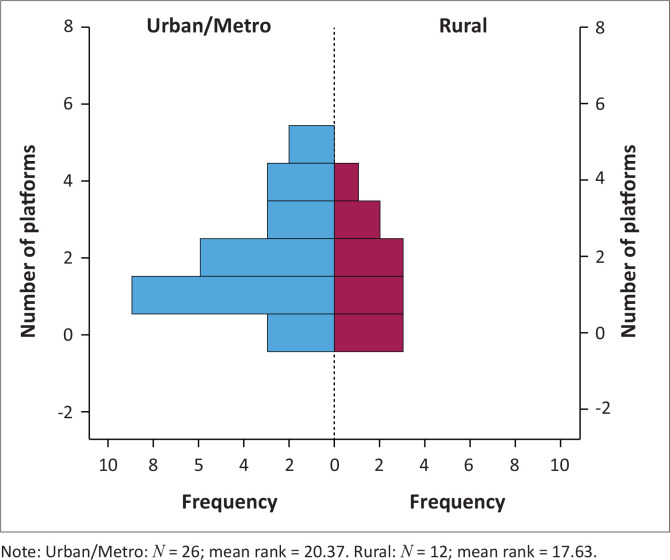
Number of social media accounts Urban/Metro versus Rural interventional radiologies.

The majority of IR practices in South Africa were in major metropolitan locations (*n* = 26, 68.4%). The median number of SM platforms used by both rural and urban practices was two platforms each, with a maximum of 4 platforms in the rural cohort and a maximum of 5 in the urban cohort. Thus, there was no statistically significant difference in the level of SM uptake between practices in urban versus non-urban and/or rural locations (Figure 2).

The median size of all general radiology practices was two radiologists and three branches.

**FIGURE 3:**
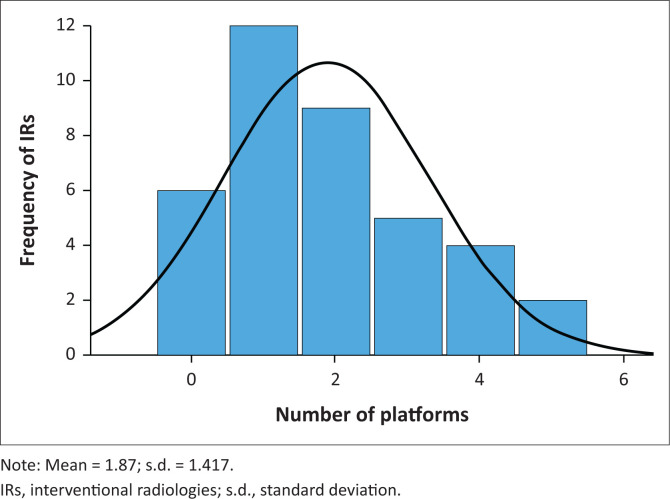
Number of social media accounts by interventional radiologies (*N* = 38).

The mean number of SM accounts was 1.87 per IR (Figure 3).

**FIGURE 4:**
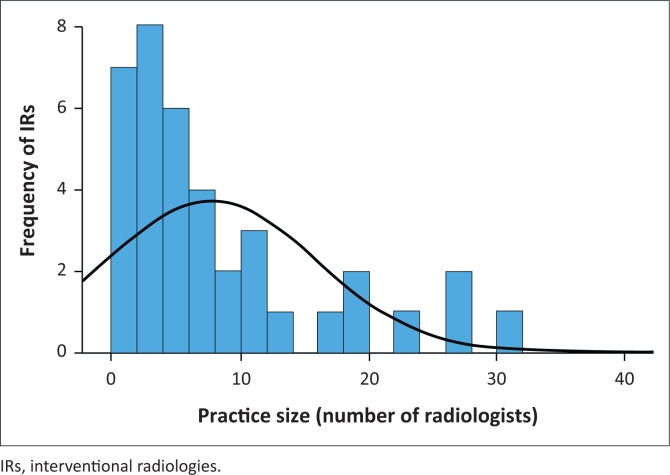
Number of General Radiologists in interventional radiology practices.

More than half (60.5%) of all IR practitioners were in practice with 5 or less radiologists (Figure 4).

**FIGURE 5:**
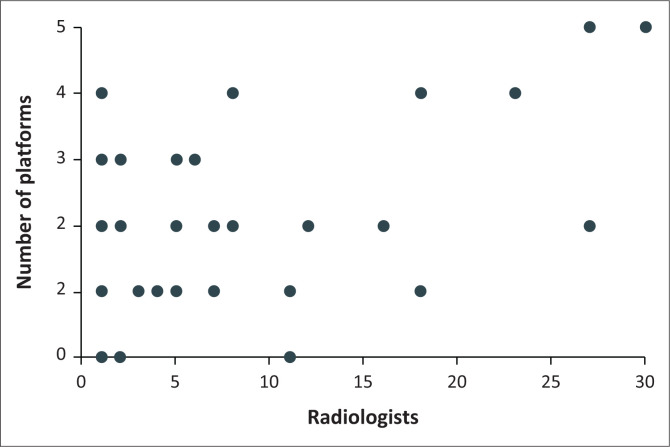
Relationship between practice size and number of social media account.

There was a positive correlation between practice size and the level of SM uptake (*p* < 0.05) using Pearson’s correlation coefficient (Figure 5).

## Discussion

Social media has become an integral part of modern life and for many people a real-time extension of their personality, ability, or ‘brand’.^15^ In January 2021, there were 4.66 billion people using the internet globally and 4.2bn of them being SM users.^16^ The number of active SM users in South Africa experienced a rapid increase of 3m users in 2020 with the total number being 25m people (41.9% of the population) in this period. The most popular platforms used in 2020 being Facebook, X (formerly Twitter), WhatsApp, YouTube, LinkedIn and Instagram.^15,16^ The South African Social Media Landscape Report 2021 published by World Wide Worx revealed that three in four businesses saw positive returns from using SM, underlining the power of SM to increase brand awareness and drive direct sales.^16^ The influence of this form of marketing has shown immense educational benefits as well.

Interventional radiology is a new and rapidly evolving sub-specialty of radiology that encompasses a wide range of minimally invasive image-guided diagnostic and therapeutic procedures in every human organ system.^8,9,10^ The advent of novel minimally invasive imaging-guided techniques has driven the growth and demand for IR services.^8^ Unfortunately, there continues to be poor awareness of IR as a medical specialty among the public and primary care providers.^10,11,17^ Recent studies have shown that patients are increasingly relying on SM to obtain medical information and discuss therapeutic options.^11,18,19^ Radiologists, particularly in their rapidly evolving technological landscape, are well placed to use SM to engage with patients and establish relevant online content.^10^ This can also improve the connectivity of geographically remote practitioners.^13,20^ Social media presents a low-resource intervention to broaden the online presence of rural practices, academic training institutions and strengthen the quality of information on programme websites.^21^

This is the first study to analyse the digital footprint and SM presence of IR practitioners in South Africa. The results of our data reveal that there is a massive shortage of IRs in South Africa with particularly poor access to these services in the rural parts of the country. Encouragingly, the majority (84.2%) of IR practitioners had an identifiable website and less than one fifth (18.8%) did not have any SM presence. The location of the practices did not reveal any statistically significant difference in SM uptake.

X (formerly Twitter) is the most popular form of SM used for healthcare communication and has become an integral mode of communication with individuals and organisations in various fields for customer relations, advocacy and public outreach.^22,23,24^ The South African Department of Health’s X (formerly Twitter) page experienced a 44% increase in the number of followers in March 2020 compared to a year prior. This surge was likely because of the coronavirus disease 2019 (COVID-19) pandemic, highlighting the utility of the platform as a source of information for the public.^7^

YouTube is a video-sharing website and is among the most used platforms in healthcare primarily targeted at patients and families. It was the second most used SM platform in South Africa in 2021.^16^ In a recent survey evaluating the public awareness of IR, most respondents preferred to learn more about the specialty by viewing short educational videos on websites such as YouTube.^11^ Unfortunately, none of the IR practices we analysed had a presence on X (formerly Twitter) and only 5.3% had a YouTube account. This represents a missed opportunity for South African IR.

Most IR practitioners in South Africa were in practice with five or fewer radiologists; however, there was a poorer SM uptake from the smaller practices in our study. This might reflect wider resource availability in larger practices allowing for greater investment in their SM presence.

### Limitations

Some limitations to this study can be expected because of a lack of a national registry of South African IRs, and it is unlikely that all practitioners’ SM accounts were identified. The online search was performed on the author’s personal computer, and results may have been influenced by inherent algorithmic bias. Finally, the study did not evaluate the manner and frequency of the SM accounts used. Nonetheless, this represents the digital perspective of the ordinary South African patient, medical students and colleagues seeking to learn and engage with the specialty. Previous studies investigated the usage of SM by South African dentists.^25^ However, these relied on self-reporting of use. This study was an audit of a professional healthcare service based on online information available to the public and is the first to evaluate SM use by a specified South African medical cohort through independent search and data collection.

## Conclusion

This study highlights the overall lack of radiologists, especially IRs in South Africa. Furthermore, it reflects the wide disparities in access to these services in the rural parts of the country. Our analysis demonstrates that the majority of South African IRs have a digital presence on at least one SM platform with a significantly wider presence seen in larger radiology practices. The poor participation on X (formerly Twitter) and YouTube represents a missed opportunity for the specialty and increased participation on these platforms particularly from smaller radiology practices may enhance their visibility to patients and referrers. The use of visual mediums, such as YouTube, is particularly valuable in radiology and the robust academic discussions on platforms such as X (formerly Twitter) present additional pathways of communication that South African IRs can leverage.

### Recommendations

The South African IR community could leverage the potential of SM platforms to improve public awareness of their services to a wider audience.

We recommend future research directions to explore the levels of SM engagement by medical practitioners and the impact of SM on referral patterns and patient experience.
